# Intimate partner violence and nutritional status among nepalese women: an investigation of associations

**DOI:** 10.1186/s12905-020-00991-x

**Published:** 2020-06-17

**Authors:** Ramesh P. Adhikari, Subash Yogi, Ajay Acharya, Kenda Cunningham

**Affiliations:** 1 Suaahara II, Helen Keller International Nepal, Patan, Lalitpur, Nepal; 2 Suaahara II, Care International, Lalitpur, Nepal; 3 Suaahara II, FHI360, Kathmandu, Nepal; 4 London School of Hygiene and Tropical Medicine, London, England

**Keywords:** Intimate partner violence, Nutrition, Underweight, Anemia, Nepal

## Abstract

**Background:**

Malnutrition among women in Nepal persists as a major public health burden. Global literature suggests that domestic violence may have a negative impact on women’s nutritional status. The contribution of intimate partner violence (IPV) to increased stress levels, poor self-care including the consumption of less food and, in turn, malnutrition has been documented. However, there is little empirical evidence on IPV and its relationship with women’s nutritional status in Nepal and thus, this paper assesses these associations.

**Methods:**

We used data on non-pregnant married women (*n =* 3293) from the 2016 Nepal Demographic and Health Survey (NDHS). The primary exposure variable was whether the women had ever experienced physical, sexual, or emotional violence or controlling behaviours by a current or former partner, based on her responses to the NDHS domestic violence questions. The primary outcome variables were three indicators of malnutrition: under-weight (BMI < 18.5), over-weight (BMI > 25), and anemia (Hb < 11.0 g dL). We used logistic and multinomial regression models, adjusted for potential socio-demographic and economic confounders, as well as clustering, to examine associations between IPV exposure and malnutrition.

**Results:**

Approximately 44% of women had experienced at least one of the four types of IPV. Among them, around 16, 25%. and 44% were underweight, overweight, or anemic, respectively, compared to 13, 29, and 35% of women never exposed to IPV. We did not find any associations between underweight and any of the four types of IPV. Overweight was associated with physical violence (adjusted RRR = 0.67, *P <* 0.01, CI = 0.50–0.88) and severe physical violence (adjusted RRR = 0.53, *P <* 0.05, CI = 0.32–0.88) Controlling behaviors were associated with anemia (adjusted RRR = 1.31, *P <* 0.01, CI = 1.11–1.54).

**Conclusions:**

Among married Nepalese women, physical violence appears to be a risk factor for one’s weight and controlling behaviors for one’s anemia status. Additional, rigorous, mixed-methods research is needed to understand the reporting of IPV and what relationships do or do not exist between IPV experience and nutrition both in Nepal and in other settings.

## Background

Intimate partner violence (IPV) against women is increasingly recognized as a public health concern as it has several consequences on women’s physical and psychosocial wellbeing. IPV includes physical, sexual, and emotional violence by a current or former partner. Global estimates show that around 30% of women who have been in a relationship have experienced violence by an intimate partner, with exposure to IPV relatively higher (38%) in South-East Asia than other regions of the world [1]. Similarly, a survey conducted in 10 different countries from 2000 to 2003 showed women’s exposure to IPV to ranges from 15 to 71% [2]. Associations between IPV prevalence and various household demographic and contextual factors, including socio-economic status have also been documented [3–6]. A recent study based on 42 demographic and health surveys from low- and middle- income countries (LMIC), revealed that about one in three women experience IPV at some point during their life [7].

IPV has negative ramifications on women’s physical and mental health; depression triggered by IPV, for example, can in turn affect a women’s ability to care for herself [1, 8, 9]. Although it seems likely that IPV has an impact on the nutritional status of affected women, studies on the links between IPV and women’s nutritional status, particularly in LMICs are limited [3]. Available literature suggests that experiencing violence could influence one’s nutritional status in various ways. For example, IPV could increase depression and stress levels [10, 11] which could result in women consuming fewer or more calories and in turn, being over or underweight. IPV may also increase a woman’s risk-taking behaviors (e.g. smoking, drug s or alcohol abuse) which in turn, would influence her self-care, dietary intake and nutritional status [12, 13]. An analysis using data from the 1998–1999 India family health survey showed that mothers who experience domestic violence multiple times in a year are more likely to be underweight and anemic, even after controlling for socio-economic and demographic factors [4].. A study in Bangladesh indicated that women of reproductive age (WRA) who experience physical or sexual violence are more likely to be underweight, with body mass index (BMI) less than 18.5 kg/m^2^ after controlling for the effect of age, education, occupation and other potentially confounding factors [3]..

In Nepal, malnutrition among WRA is a serious public health: two in every five (41%) are anemic, while 17% are underweight (BMI < 18.5 kg/m^2^) and 22% overweight or obese (BMI > 25 kg/m^2^). Prevalence rates vary by region of the country, socio-economic status, and other factors. The 2016 Nepal Demographic and Health Survey (NDHS) also highlighted that 26% of ever married WRA have experienced IPV at some point [14]. In Nepal, because of patriarchal norms and socio-cultural practices, women may face discrimination and even shame and social isolation if they share domestic problems and seek support from others. Thus, due to self-blame and stigma, IPV may be under reported in surveys in Nepal [1, 15–17] . There are no studies to date, however, looking at whether there’s an association between experiencing IPV and nutritional status in Nepal. Therefore, this study assesses associations between IPV and women’s nutritional status, including underweight, overweight/obesity, and anemia in Nepal.

## Methods

This paper uses data from the 2016 NDHS, a nationally representative cross-sectional household survey. This dataset includes information on a wide variety of health topics, as well as socio-economic and demographic factors; additional information, such as women’s experience with domestic violence, was collected among sub-samples. The sampling details for this survey have been documented in the full NDHS report [14]. Among the 12,862 WRA included in the survey, the domestic violence module was administered to 4444 women. For this analysis, we included the 3310 women (among the 4444 women) who were ever married, but neither currently pregnant nor had given birth in the previous 2 months. Some cases were further excluded because their BMI measurement was an outlier (*N =* 7) or they had refused to have their biomarker data collected (*N =* 10). Thus, the final sample size for analyses done for this paper was *N =* 3293 [14].

Three indicators of women’s nutritional status were used as outcome variables: underweight (body mass index [BMI] less than 18.5), overweight/obesity (BMI of 25 or more), and anemia (hemoglobin level of less than 11 g per deciliter).

IPV, the primary exposure variable, was measured in two different ways based on 13 questions related to emotional, physical, and sexual violence and 5 questions related to controlling behaviours. Questions on emotional violence asked the woman if she had ever been humiliated in front of others; threatened or had someone close to her threatened with harm; or insulted or made to feel bad about herself. Questions on physical violence included asking the woman if a partner had ever pushed, shaken, or thrown something at her; slapped or twisted her arm; punched her with a fist or something that could hurt; kicked or dragged her; tried to strangle or burn her; or attacked her with a knife, gun, or other weapon or threatened to do so. Sexual violence questions included whether she had been forced to engage or threatened by sexual intercourse and acts. Questions related to controlling behaviours included whether she felt that her husband/partner was jealous or angry if she talked to other men; frequently accused her of being unfaithful; did not permit her to meet her female friends; tried to limit her contact with her family; or insisted on knowing where she is at all times. For the first measurement of IPV, responses to each of the 13 questions related to emotional, physical, and sexual violence and 5 questions related to controlling behaviours were combined to generate a dichotomous variable denoting “any experience of IPV including controlling behaviours “ if she answered “yes” to any of the 18 questions. For the second measurement of IPV, we focused only on the 13 physical, emotional and sexual violence questions. Additionally, the severity of physical violence was measured based on none (never experienced physical violence) moderate (if a partner had ever pushed, shaken, or thrown something at her or slapped her) or severe (if the partner had ever twisted her arm or punched her with a fist or something that could hurt; kicked or dragged her; tried to strangle or burn her; or attacked her with a knife, gun, or other weapon or threatened to do so).

Potentially confounding socio-demographic and economic factors, selected based on knowledge of the local context and prior studies on nutrition and IPV in LMICs, particularly in South Asia were included in the adjusted models: the respondent woman’s age in years and years of formal schooling as well as household size, caste/ethnicity (defined as Dalit, Muslim, Janajati, other terai caste, Brahmin/Chhetri, and others), wealth status (using DHS wealth quintiles), and place of residency (urban and rural).

To explore associations between IPV and malnutrition, logistic and multinomial regression models were used. We also assessed multicollinearity among the different types of IPV and then explored associations between each type of IPV and each indicator of malnutrition. The weighted sample was used to adjust for the survey design effect. To adjust for clustering, the primary sampling unit (sub-ward) was used. All data analysis was performed in Stata14.

## Results

### Characteristics of the study population

The median age of the married, non-pregnant women in this sample was 32 years and more than two-fifths (44%) of the respondents had no formal schooling. Nearly one-third of the respondents belonged to the Brahmin/Chhetri caste/ethnic group and about three-fifths (60%) resided in urban areas of Nepal. Among the respondent women, around 44% reported to have experienced at least one type of IPV at some point in their life; around 14% were underweight, 27% overweight/obese and 39% anemic (Table 1).

**Table 1:** Socio-demographic characteristics, exposure to intimate partner violence, and nutritional status of the respondent women (*N* = 3293)

	N	%
Woman’s age (in completed years)		
15–19	170	5.2
20–24	512	15.5
25–29	601	18.3
30–34	613	18.6
35–39	579	17.6
40–44	481	14.6
45–49	336	10.2
Woman’s education (by years of formal schooling)		
No school	1458	44.3
1–5 years school	568	17.2
6–9 years school	678	20.6
10 and above years of school	589	17.9
Family size		
Less than 5	1473	44.7
5 and above	1820	55.3
Caste/ethnicity		
Dalit	433	13.2
Muslim	156	4.7
Janajati	1023	31.1
Other *terai* caste	495	15.0
Brahmin/Chhetri	1016	30.9
Other	170	5.2
Place of residence		
Urban	1978	60.1
Rural	1315	39.9
Wealth quintile		
Poorest	578	17.6
Second poorest	650	19.7
Middle	698	21.2
Second richest	707	21.5
Richest	660	20.0
Ever experienced intimate partner violence (*N =* 3293)		
Physical violence		23.0
Emotional violence		12.2
Sexual violence		7.0
Controlling behaviours		33.7
Overall violence (excluding controlling behaviours)		26.4
Overall violence (including controlling behaviours)		43.8
Severity of physical violence (*N =* 3293)		
None		77.0
Moderate		13.0
Severe		10.0
Nutritional status (*N =* 3293)		
Underweight		14.2
Overweight		27.1
Anemic		39.1

### Bivariate analysis

The prevalence of having ever experienced IPV was around 42–50% for each age category, but the prevalence was highest among women aged 35–39 years (49.5%). We found a much higher prevalence of having experienced IPV among women who had no schooling (49%) than those with the highest levels of schooling (34.3%) (*P <* 0.001). Fewer Brahmin/Chettri women reported IPV (31%) than any other caste/ethnicity group (*P <* 0.001). Underweight women tended to be younger (*P <* 0.001), have fewer years of schooling (*P <* 0.001), live with larger families (*P <* 0.001), and reside in rural areas (*P <* 0.001). Overweight women tended to be older (*P <* 0.001), educated with at least some formal schooling (*P <* 0.001), reside with wealthier families (*P <* 0.001) and in urban areas (*P <* 0.001). The differences among women with anemia vs. those without were not as drastic. Anemia, however, seems to be a greater problem for terai caste groups and Muslims (who are also heavily concentrated in the terai) (*P <* 0.001) (Table 2).

**Table 2:** Women’s nutritional status by exposure to intimate partner violence, and both by socio-demographic and economic characteristics (*N* = 3293)

	Ever experienced IPV (including controlling behaviours)	***P*** value	Under-weight	***P*** value	Over-weight	***P*** value	Anemic	***P*** value	N
	%		%		%		%		
IPV (including controlling behaviours)									
Yes			15.9	0.042	24.6	0.055	44.0	< 0.001	1442
No			13.0		29.1		35.3		1851
IPV (excluding controlling behaviours)									
Yes			18.1	0.001	21.5	0.002	44.1	0.011	869
No			12.9		29.2		37.3		2424
Types of violence									
*Physical violence*									
Yes			18.6	< 0.001	19.1	< 0.001	44.4	0.009	757
No			12.9		29.6		37.5		2536
*Emotional violence*									
Yes			18.7	0.011	25.1	0.502	39.0	0.965	402
No			13.6		27.4		39.1		2891
*Sexual violence*									
Yes			18.6	0.065	23.5	0.270	45.3	0.081	231
No			13.9		27.4		38.6		3062
*Controlling behaviours*									
Yes			16.1	0.052	24.9	0.152	44.8	< 0.001	1110
No			13.3		28.3		36.2		2183
Severity of physical violence									
None			12.9		29.5		37.5		2536
Moderate			15.9	< 0.001	21.7	< 0.001	45.9	0.033	428
Severe			22.1		15.8		42.4		329
Woman’s age (in completed years)									
15–19	44.6	0.230	26.8	< 0.001	4.7	< 0.001	40.5	0.790	170
20–24	42.3		18.8		14.1		41.7		512
25–29	44.7		15.3		26.0		40.2		601
30–34	42.5		10.6		34.2		39.6		613
35–39	49.5		9.3		31.4		38.1		579
40–44	40.0		16.2		30.2		37.6		481
45–49	41.6		11.4		35.8		35.4		336
Woman’s education (by years of formal schooling)									
No school	49.0	< 0.001	18.9	< 0.001	19.9	< 0.001	41.1	0.498	1458
1–5 years of school	46.8		12.5		32.2		38.3		568
6–9 years of school	38.0		10.3		31.8		37.5		678
10 and above years of school	34.3		9.0		35.0		36.7		589
Family size									
Less than 5	42.8	0.431	9.8	< 0.001	31.5	< 0.001	36.4	< 0.05	1473
5 and above	44.5		17.9		23.6		41.3		1820
Caste/ethnicity									
Dalit	54.9	< 0.001	20.8	< 0.001	22.7	< 0.001	38.9	< 0.001	433
Muslim	59.1		31.3		16.6		52.3		156
Janajati	41.1		10.6		31.8		35.5		1023
Other terai caste	59.7		22.8		14.9		54.4		495
Brahmin/Chhetri	30.9		9.8		28.2		36		1016
Other	47.3		5.3		49.8		23.4		170
Place of residence									
Urban	44.7	0.434	11.5	< 0.001	32.9	< 0.001	37.2	0.118	1978
Rural	42.3		18.4		18.5		41.9		1315
Wealth quintile									
Poorest	36.2	< 0.001	17.1	< 0.001	11.3	< 0.001	28.1	< 0.001	578
Second poorest	43.0		18.0		19.4		37.8		650
Middle	49.8		16.7		15.2		48.4		698
Second richest	49.7		15.9		30.2		44.8		707
Richest	38.2		3.7		58.0		34.0		660

Among respondent women, those who had ever experienced IPV were more likely to be underweight (16% vs. 13%; *P <* 0.05) and to have had anemia (44% vs 35%) (*P <* 0.001) compared to those who had never experienced IPV. Those who had experienced severe physical violence also had a higher prevalence of being underweight than those who had experienced moderate or no physical violence (22% vs. 16% vs. 13%; *P <* 0.001) (Table 2).

### Multivariate analysis

Table 3 provides the results from analyses of adjusted associations between a woman having ever experienced IPV (including and excluding controlling behaviours) and being malnourished including underweight, overweight and anemia, respectively. The adjusted multinomial logistic regression model indicated that having experienced IPV, regardless of whether the definition includes or excludes controlling behaviors, was not associated with an increased or decreased risk of being underweight or overweight. The adjusted logistic regression model results, however indicated that exposure to IPV, when including controlling behaviours in the definition, was associated with increased odds of anemia.

**Table 3:** Associations of intimate partners violence and nutritional status, measured by Body Mass Index (BMI) and anemia among married Nepalese women of reproductive age (*N* = 3293)

	Nutritional status with IPV (excluding controlling behaviours)			Nutritional status with IPV (including controlling behaviours)		
	Normal Vs. Underweight (BMI < 18.5)	Normal Vs. Overweight (BMI > 25.0)	Anemia (Hemoglobin < 11.0 g/dl)	Normal Vs. Underweight (BMI < 18.5)	Normal Vs. Overweight (BMI > 25.0)	Anemia (Hemoglobin < 11.0 g/dl)
	Adj. RRR (95%, CI)	Adj. RRR (95%, CI)	Adj. OR (95%, CI)	Adj. RRR (95%, CI)	Adj. RRR (95%, CI)	Adj. OR (95%, CI)
Experience of intimate partner violence (ref: no)						
Yes	1.12 (0.86–1.46)	0.77 (0.57–1.03)	1.21 (0.98–1.52)	0.97 (0.76–1.24)	0.84 (0.66–1.08)	1.31** (1.11–1.54)
Woman’s age	0.98* (0.96–0.99)	1.05*** (1.04–1.07)	0.99 (0.98–1.01)	0.98* (0.96–0.99)	1.05*** (1.03–1.07)	0.99 (0.98–1.01)
Woman’s schooling years	0.96* (0.92–0.99)	1.03 (0.99–1.06)	0.99 (0.97–1.02)	0.96* (0.92–0.99)	1.03 (1.00–1.06)	0.99 (0.97–1.02)
Family size	1.04 (0.99–1.08)	0.94 (0.87–1.01)	1.00 (0.96–1.04)	1.03 (0.99–1.08)	0.94 (0.87–1.01)	1.00 (0.96–1.04)
Caste/ethnicity (ref: Dalit)						
Muslim	1.6 (0.92–2.86)	0.67 (0.57–1.03)	1.42 (0.85–2.38)	1.6 (0.92–2.87)	0.67 (0.33–1.35)	1.42 (0.86–2.37)
Janajati	0.55* (0.37–0.81)	1.10 (0.79–1.52)	0.90 (0.64–1.27)	0.54** (0.36–0.80)	1.10 (0.80–1.52)	0.91 (0.65–1.28)
Other terai caste	1.00 (0.62–1.62)	0.44*** (0.27–0.72)	1.53 (0.98–2.43)	1.00 (0.62–1.63)	0.44*** (0.27–0.72)	1.53 (0.96–2.42)
Brahmin/Chhetri	0.54* (0.37–0.79)	0.63** (0.45–0.89)	0.98 (0.70–1.37)	0.53** (0.36–0.78)	0.63** (0.45–0.89)	1.00 (0.71–1.40)
Other	0.46 (0.18–1.19)	1.04 (0.65–1.69)	0.51* (0.27–0.96)	0.46 (0.18–1.18)	1.04 (0.64–1.68)	0.51* (0.27–0.96)
Place of residence (ref: urban)						
Rural	1.14 (0.84–1.55)	0.96 (0.74–1.25)	1.17 (0.90–1.51)	1.14 (0.84–1.54)	0.96 (0.74–1.25)	1.18 (0.91–1.52)
Wealth quintiles (ref: poorest)						
Second poorest	1.14 (0.83–1.58)	1.97*** (1.38–2.83)	1.52** (1.14–1.03)	1.15 (0.83–1.59)	1.98*** (1.38–2.84)	1.51** (1.14–1.02)
Middle	0.79 (0.54–1.15)	1.63* (1.09–2.44)	2.12*** (1.57–2.85)	0.80 (0.54–1.17)	1.63* (1.09–2.44)	2.09*** (1.56–2.81)
Second richest	1.02 (0.66–1.56)	4.23*** (2.91–6.13)	1.93*** (1.40–2.66)	1.02 (0.66–1.58)	4.28*** (2.94–6.24)	1.89*** (1.38–2.60)
Richest	0.47* (0.25–0.89)	9.50*** (6.08–14.84)	1.52* (1.05–2.21)	0.47* (0.25–0.90)	9.58*** (6.13–14.96)	1.50* (1.08–2.18)

* *p <* 0.05; ** *p* < 0.01; *** *p* < 0.001; *Adj. RRR* Adjusted Relative Risk Ratio, *Adj. OR* Adjusted Odds Ratio, *CI* Confidence Interval, *ref.* Reference Category

Some of the socio-economic and demographic factors, such as wealth were associated with one’s nutritional status: women from less wealthy households had an increased risk of being underweight (RRR 0.47, CI: 0.25–0.89) whereas those from wealthier households had an increased risk of being overweight (RRR 9.50, CI: 6.08–14.84). Likewise, having a lower level of education was associated with an increased risk of being underweight (RRR 0.96, CI: 0.92–0.99).

Using adjusted multinomial logistic regression models, we show results for each specific type of IPV and overweight and underweight versus anemia, respectively (Tables 4 and 5) We did not find any association between any of the 4 types of IPV and underweight. Women exposed to any physical violence had a decreased risk of being overweight/obese, but there was no association for the other 3 types of IPV. Adjusted odds ratios indicated that, only controlling behaviours and none of the other specific types of IPV were associated with an increased risk of being anemic.

**Table 4:** Associations between different types of intimate partner violence and nutritional status, measured by Body Mass Index (BMI) among married Nepalese women of reproductive age (*N* = 3293)

	BMI with physical violence		BMI with sexual violence		BMI with emotional violence		BMI with controlling behaviours	
	Normal Vs. Underweight	Normal Vs. Overweight	Normal Vs. Underweight	Normal Vs. Overweight	Normal Vs. Underweight	Normal Vs. Overweight	Normal Vs. Underweight	Normal Vs. Overweight
	Adj. RRR (95%, CI)	Adj. RRR (95%, CI)	Adj. RRR (95%, CI)	Adj. RRR (95%, CI)	Adj. RRR (95%, CI)	Adj. RRR (95%, CI)	Adj. RRR (95%, CI)	Adj. RRR (95%, CI)
Experience of intimate partner violence (ref: no)								
Yes	1.06 (0.80–1.39)	0.67** (0.50–0.88)	1.24 (0.85–1.83)	0.83 (0.49–0.86)	1.30 (0.98–1.83)	0.97 (0.65–1.44)	1.01 (0.79–1.29)	0.89 (0.70–1.14)
Woman’s age	0.98* (0.96–0.99)	1.05*** (1.04–1.07)	0.98* (0.96–0.99)	1.05*** (1.03–1.06)	0.98* (0.96–0.99)	1.05*** (1.04–1.06)	0.98* (0.96–0.99)	1.05*** (1.03–1.06)
Woman’s schooling years	0.96* (0.92–0.99)	1.03 (0.99–1.06)	0.96* (0.92–0.99)	1.03 (1.00–1.07)	0.96* (0.92–0.99)	1.03 (1.00–1.07)	0.96* (0.92–0.99)	1.03 (1.00–1.07)
Family size	1.04 (0.99–1.08)	0.94 (0.88–1.01)	1.04 (0.99–1.08)	0.94 (0.87–1.03)	1.04 (0.99–1.09)	0.94 (0.87–1.01)	1.03 (0.99–1.08)	0.94 (0.87–1.01)
Caste/ethnicity (ref: Dalit)								
Muslim	1.62 (0.92–2.86)	0.67 (0.33–1.36)	1.62 (0.92–2.85)	0.67 (0.33–1.38)	1.65 (0.93–2.90)	0.67 (0.33–1.36)	1.63 (0.92–2.87)	0.67 (0.33–1.35)
Janajati	0.55* (0.37–0.81)	1.08 (0.78–1.49)	0.55** (0.37–0.80)	1.13 (0.82–1.56)	0.55** (0.37–0.81)	1.13 (0.81–1.56)	0.54** (0.37–0.80)	1.12 (0.81–1.54)
Other terai caste	1.00 (0.62–1.62)	0.44*** (0.27–0.72)	1.00 (0.62–1.62)	0.44*** (0.27–0.73)	1.01 (0.62–1.64)	0.44*** (0.27–0.72)	1.00 (0.62–1.62)	0.44*** (0.27–0.73)
Brahmin/Chhetri	0.54* (0.36–0.79)	0.62** (0.44–0.87)	0.53* (0.36–0.78)	0.65* (0.46–0.92)	0.54** (0.37–0.80)	0.66* (0.47–0.92)	0.53** (0.36–0.78)	0.65* (0.47–0.91)
Other	0.46 (0.18–1.19)	1.04 (0.65–1.69)	0.46 (0.18–1.19)	1.04 (0.64–1.68)	0.45 (0.17–1.16)	1.05 (0.65–1.69)	0.46 (0.18–1.18)	1.05 (0.65–1.69)
Place of residence (ref: urban)								
Rural	1.14 (0.84–1.55)	0.96 (0.74–1.24)	1.14 (0.84–1.55)	0.97 (0.75–1.25)	1.14 (0.84–1.55)	0.97 (0.75–1.26)	1.14 (0.84–1.55)	0.96 (0.74–1.25)
Wealth quintiles (ref: poorest)								
Second poorest	1.14 (0.83–1.58)	1.98*** (1.38–2.83)	1.14 (0.84–1.55)	1.96*** (1.37–2.81)	1.15 (0.83–1.60)	1.97*** (1.38–2.81)	1.15 (0.83–1.59)	1.97*** (1.38–2.82)
Middle	0.79 (0.54–1.15)	1.63* (1.09–2.44)	0.79 (0.54–1.15)	1.62* (1.08–2.41)	0.79 (0.54–1.15)	1.62* (1.08–2.41)	0.79 (0.54–1.16)	1.62* (1.09–2.42)
Second richest	1.02 (0.66–1.56)	4.20*** (2.89–6.09)	1.02 (0.66–1.56)	4.21*** (2.91–6.10)	1.02 (0.66–1.57)	4.21*** (2.91–6.08)	1.02 (0.66–1.57)	4.25*** (2.93–6.18)
Richest	0.47* (0.25–0.90)	9.44*** (6.05–14.73)	0.47* (0.25–0.90)	9.51*** (6.11–14.83)	0.48* (0.25–0.90)	9.50*** (6.10–14.78)	0.47* (0.25–0.90)	9.53*** (6.11–14.85)

* *p* < 0.05; ** *p* < 0.01; *** *p* < 0.001; *Adj. RRR* Adjusted Relative Risk Ratio, *CI* Confidence Interval, *ref.* Reference Category

**Table 5:** Associations between different types of intimate partner violence and nutritional status, measured by anemia among married Nepalese women of reproductive age (*N* = 3293)

	Anemia with physical violence	Anemia with sexual violence	Anemia with emotional violence	Anemia with controlling behaviours
	Adj. OR (CI)	Adj. OR (CI)	Adj. OR (CI)	Adj. OR (CI)
Experience of intimate partner violence (ref: no)				
Yes	1.20 (0.97–1.49)	1.26 (0.92–1.71)	0.97 (0.73–1.29)	1.31* (1.09–1.58)
Woman’s age	0.99 (0.98–1.01)	0.99 (0.98–1.01)	0.99 (0.98–1.01)	0.99 (0.98–1.01)
Woman’s schooling	0.99 (0.97–1.02)	0.99 (0.97–1.02)	0.99 (0.97–1.02)	0.99 (0.97–1.02)
Family size	1.00 (0.96–1.04)	1.00 (0.96–1.04)	1.00 (0.96–1.04)	1.00 (0.96–1.04)
Caste/ethnicity (ref: Dalit)				
Muslim	1.42 (0.85–2.37)	1.41 (0.85–2.36)	1.42 (0.85–2.37)	1.43 (0.87–2.36)
Janajati	0.9 (0.64–1.26)	0.88 (0.63–1.24)	0.88 (0.62–1.24)	0.90 (0.64–1.26)
Other terai caste	1.53 (0.96–2.42)	1.54 (0.97–2.42)	1.53 (0.97–2.42)	1.51 (0.96–2.39)
Brahmin/Chhetri	0.97 (0.69–1.36)	0.95 (0.68–1.33)	0.94 (0.67–1.32)	0.98 (0.70–1.37)
Other	0.51* (0.27–0.96)	0.51* (0.27–0.96)	0.51* (0.27–0.95)	0.51* (0.27–0.96)
Place of residence (ref: urban)				
Rural	1.16 (0.90–1.51)	1.16 (0.90–1.50)	1.16(0.89–1.50)	1.18 (0.91–1.51)
Wealth quintiles (ref: poorest)				
Second poorest	1.52** (1.14–2.03)	1.53** (1.15–2.05)	1.53** (1.14–2.04)	1.53** (1.14–2.03)
Middle	2.12*** (1.57–2.86)	2.13*** (1.58–2.87)	2.13*** (1.58–2.88)	2.11*** (1.58–2.83)
Second richest	1.94*** (1.41–2.66)	1.93*** (1.40–2.65)	1.93*** (1.41–2.65)	1.89*** (1.38–2.60)
Richest	1.52* (1.05–2.22)	1.51* (1.04–2.20)	1.51* (1.04–2.20)	1.51* (1.04–2.18)

* *p* < 0.05; ** *p* < 0.01; *** *p* < 0.001; *Adj. OR* Adjusted Odds Ratio, *CI* Confidence Interval, *ref.* Reference Category

Table 6 reports that we found no association between the severity of physical violence and risk of being underweight or anemic. Women exposed to severe physical violence, however, had a decreased risk of being overweight/obese.

**Table 6:** Associations between severity of physical intimate partner violence and nutritional status, measured by Body Mass Index (BMI) and Anemia among married Nepalese women of reproductive age (*N* = 3293)

	Normal Vs. Underweight (BMI < 18.5)	Normal Vs. Overweight (BMI > 25.0)	Anemia (Hemoglobin < 11.0 g/dl)
	Adj. RRR (95%, CI)	Adj. RRR (95%, CI)	Adj. OR (95%, CI)
Severity of physical violence (ref: no)			
Moderate	0.89 (0.61–1.23)	0.77 (0.57–1.06)	1.23 (0.96–1.59)
Severe	1.29 (0.89–1.86)	0.53* (0.32–0.88)	1.17 (0.86–1.58)
Woman’s age	0.98* (0.96–0.99)	1.05*** (1.04–1.07)	0.99 (0.98–1.01)
Woman’s schooling	0.96* (0.92–0.99)	1.03 (0.99–1.06)	0.99 (0.97–1.02)
Family size	1.04 (0.99–1.09)	0.94 (0.88–1.01)	1.00 (0.96–1.04)
Caste/ethnicity (ref: Dalit)			
Muslim	1.62 (0.92–2.85)	0.68 (0.33–1.39)	1.42 (0.85–2.37)
Janajati	0.55** (0.37–0.81)	1.09 (0.79–1.50)	0.9 (0.64–1.26)
Other terai caste	1.01 (0.62–1.65)	0.44*** (0.27–0.71)	1.52 (0.96–2.42)
Brahmin/Chhetri	0.54** (0.37–0.80)	0.62** (0.44–0.87)	0.97 (0.69–1.36)
Other	0.46 (0.18–1.19)	1.06 (0.65–1.72)	0.51* (0.27–0.95)
Place of residence (ref: urban)			
Rural	1.13 (0.84–1.54)	0.96 (0.74–1.24)	1.17 (0.90–1.51)
Wealth quintile (ref: poorest)			
Second poorest	1.16 (0.83–1.61)	1.95*** (1.36–2.78)	1.52** (1.14–2.03)
Middle	0.80 (0.54–1.17)	1.62* (1.08–2.41)	2.12*** (1.58–2.85)
Second richest	1.03 (0.66–1.59)	4.14*** (2.86–6.00)	1.93*** (1.41–2.65)
Richest	0.48* (0.25–0.91)	9.39*** (6.02–14.63)	1.52* (1.05–2.21)

* *p* < 0.05; ** *p* < 0.01; *** *p* < 0.001; *Adj. RRR* Adjusted Relative Risk Ratio, *Adj. OR* Adjusted Odds Ratio, *CI* Confidence Interval, *ref*. Reference Category

## Discussion

This paper generates evidence on associations between IPV and women’s nutritional status in Nepal, based on a nationally representative data set. Around 44% of women had ever experienced emotional, physical, or sexual violence or controlling behaviours from their spouse/partner. Among the sample population, malnutrition was also a problem: 14% were underweight, 27% overweight/obese and 39% anemic. In final, adjusted models, we found no association of IPV, regardless of whether the definition included or excluded controlling behaviours, on underweight and overweight. IPV, when defined to include controlling behaviours, however, was associated with anemia. Additionally, none of the specific types of violence was associated with being underweight, but exposure to physical IPV was associated with a decreased risk of being overweight/obese. Likewise, the severity of physical violence was not associated with being underweight but the greater the severity of physical IPV, the lower the risk of being overweight/obesity.

Some of the null findings in this study may result from women’s under-reporting of IPV due to self-blame, shame and stigma, and social desirability bias [1, 17]. The tools used to measure domestic violence were developed and validated by WHO multi-country team and pre-tested in six countries (Bangladesh, Brazil, Namibia, Samoa, Thailand and the United Republic of Tanzania). The validation suggests that the instrument should provide reliable and valid measures for violence and is thus widely used in DHS globally. This tool, however, has not been validated in Nepal and thus may not accurately capture IPV experience and reporting in this context [18]. Furthermore, the NDHS module asks about IPV throughout one’s life, whereas the nutritional measurement is at the time of the survey; thus, if the violence occurred at a much earlier point in life, it is reasonable to assume that it may not affect one’s nutritional status as many factors throughout one’s life combine to influence one’s nutritional status at any given time. Finally, the lack of associations between IPV and nutritional status among Nepalese women could also be because in this context, IPV may not result in food being used as a control mechanism or that there is any relationship between suffering from IPV and being denied access to foods and services, which are important for nutritional well-being.

The association we found between controlling behaviors, but not other types of IPV, and anemia could suggest that controlling behaviors generate more prolonged psychological stress. This is a known risk factor for oxidative stress, which contributes to anemia [19]. We hypothesize, therefore, that chronic stress generated from experiencing controlling behaviors may be a reason these women were more at risk of being anemic [20]. The association found between physical violence and a decreased risk of overweight/obesity was consistent with the results of a cross-sectional population based study in Brazil which suggested that physical IPV was negatively associated with BMI [21]. On the other hand, some studies have found physical and non-physical IPV increased the risk of overweight/obesity of women [22–24]. These divergent global findings suggest a need for further research.

Ackerson and Subramanian (2008) reported that domestic violence had a significant positive association with underweight and anemia among married women in India [4], yet their results showed that the associations were only significant for underweight when IPV experience has happened in the 12 months prior to the survey and for anemia only when IPV was experienced multiple times in the 12 months prior to the survey. They also found no significant association between underweight and anemia and violence experienced more than 1 year ago, which is similar to our measurement of ever experienced IPV. Another study conducted in Bangladesh, however, reported that women who had experienced IPV ever had 1.24 times greater odds of being underweight [3]. Although this is inconsistent with our findings, there are several reasons why comparison with this study is challenging. The prevalence of violence (53% vs. 26%) and underweight (28% vs 14%) were both substantially greater in the Bangladesh study compared to our study, which may indicate that the study had greater power to detect a relationship. The Bangladesh study also only included physical violence and sexual violence, whereas we included physical, sexual and emotional violence in our definition of IPV. Finally, each study used slightly different confounders which may also explain differing results: wealth, for instance, was not adjusted for in the Bangladesh study and we found it to be a highly significant confounder in our analyses.

## Conclusion

Our analyses were based on cross-sectional survey data, making causal assessment of the relationships between an individual experiencing IPV and her nutritional status impossible. Also because of the sensitivity and social stigma relating to IPV, there is a possibility of underreporting, especially when a module like this is integrated into a much longer health survey making in-depth rapport building needed to discuss sensitive topics more challenging. Despite these limitations, this study is unique in its assessment of the associations between the experience of IPV and women’s nutritional status in Nepal, particularly looking at multiple indicators of malnutrition. The use of a nationally representative dataset is another study strength as it means the findings are generalizable at a population level. To the best of our knowledge, this is the first study to explore associations between a woman being exposed to IPV and her nutritional status in Nepal and only the third to do so ever using data from South Asia. Additional rigorous research using mixed methods is needed to understand the prevalence of IPV and why IPV is not associated with underweight, and overweight/obesity in this population, particularly given that it is associated in other South Asian contexts.

## Data Availability

The datasets used in this study are available from the corresponding author based on request.

## Abbreviations

BMI: Body Mass Index
CI: Confidence Interval
DHS: Demographic and Health Survey
IPV: Intimate Partner Violence
LMICs: Low- and Middle- Income Countries
NDHS: Nepal Demographic and Health Survey
OR: Odds Ratio
ref.: Reference Category
RRR: Relative Risk Ratio
WRA: Women of Reproductive Age
